# Second primary cancers in patients with squamous cell carcinoma of the skin

**DOI:** 10.1038/sj.bjc.6602306

**Published:** 2004-12-21

**Authors:** S K Maitra, H Gallo, C Rowland-Payne, D Robinson, H Møller

**Affiliations:** 1 Thames Cancer Registry, Division of Cancer Studies, Guy's, King's and St Thomas' School of Medicine, Capital House, 42 Weston Street, London SE1 3QD, UK; 2 The London Clinic, 149 Harley Street, London W1N 2DE, UK

**Keywords:** squamous cell, malignancy, neoplasm, skin, second primary

## Abstract

The occurrence of second primary cancers was explored in patients with squamous cell cancer of the skin (SCC). The excess incidence subsequent to SCC was mainly in cancers related to sunlight and smoking, and in lymphoproliferative malignancies, it was largest (10-fold) in salivary gland cancer.

Malignant tumours of the skin are common cancers, typically affecting fair skinned racial groups (Armstrong and Kricker, 2001). The UV component of sunlight is regarded as the most important factor leading to squamous cell cancer (SCC). Other dominant risk factors are smoking and immunosuppression. A number of studies have found significantly increased risks of various second primary cancers in SCC patients (Frisch and Melbye, 1995; Levi *et al*, 1997; Wassberg *et al*, 1999; Askling *et al*, 1999; Hemminki and Dong, 2000; Hemminki *et al*, 2001; Efird *et al*, 2002). Some general patterns can be seen from these studies. There is an increased risk for the development of subsequent skin cancers, cancers of the lip, lungs, pharynx, larynx, the salivary glands and non-Hodgkin's lymphoma.

We have examined the occurrence of primary cancer subsequent to the development of SCC.

## MATERIALS AND METHODS

Information on patients diagnosed with SCC and subsequent primary cancers was obtained from the Thames Cancer Registry (TCR). The TCR records data on the occurrence of cancer in the 14 million population of Southeast England.

Patients diagnosed with a primary SCC between 1 January 1961 and 31 December 2000 were extracted from the database. This numbered 25 731 cases (16 962 men and 8769 women). The classification of a second primary tumour excludes cases of metastasis or reoccurrence of the initial malignancy. The standardised incidence ratio (SIR) for the development of each second primary cancer was calculated by dividing the observed number by the expected number, obtained from age and sex-specific cancer incidence rates for the area. For a given subsequent cancer site, person-years at risk were calculated from the date of diagnosis of SCC to the date of first diagnosis of cancer at the specified site or to the exit date (date of death, loss to follow-up or 85th birthday, whichever was earlier). Patients diagnosed prior to 1 January 1971 were followed up actively, obtaining death information, until 31 December 1982. These were censored at this date. Patients diagnosed after 1 January 1971 were followed up through the NHS Central Registry, which provides notification of all deaths routinely to the TCR. Byar's method was used for the calculation of 95% confidence intervals for the SIR values.

## RESULTS

There were 3359 cases of second primary cancers after SCC diagnosis, of which 2567 were in male patients and 792 were in female patients (Table 1 ). The SIR for the occurrence of any second primary cancer was 1.2 in both male and female subjects.

There was an increased risk for non melanoma skin cancer (BCC was not included). Risk for malignant melanoma was significantly increased in both male and female subjects (SIR male 3.0; female 2.9). Lip cancer risk was greatly elevated (SIR male 3.5; female 7.7).

Relative risk of cancer of the pharynx was elevated, and statistically significantly so in female subjects. Risk of oesophagus cancer was increased in males, and colon cancer incidence was increased in both sexes. Salivary gland cancer risk was particularly elevated (SIR male 11.0; female 10.6).

The respiratory system in general was at an increased susceptibility to second primary cancers. Lung cancer occurrence was especially striking, not only due to the increased SIR values in both sexes (SIR male 1.3; female 1.2), but also because of the large absolute excess incidence. Lung cancer accounted for 24% of the total second primary cancer occurrence, and 28% of the excess burden of cancer in the cohort. The nasal cavity (and ear) was at a significantly increased risk in female subjects and laryngeal cancer displayed a significantly increased risk in male subjects.

There was an evident increase in occurrence of lymphoma and leukaemia. Hodgkin's disease had a raised SIR value (1.4) in males only. However, non-Hodgkin's lymphomas (comprising follicular, diffuse and other varieties) showed consistently elevated SIR values in both sexes. A significantly increased risk of development of lymphoid leukaemia was also observed (SIR male 1.7; female 1.8).

Generally, the risk of second cancer was highest within the first year following SCC diagnosis. The cancers with the highest and most sustained SIRs were lip cancer (in males), salivary gland cancer, malignant melanoma of the skin, laryngeal cancer (in males), non-Hodgkin's lymphoma (diffuse and other varieties excluding follicular) and lymphoid leukaemia. However, salivary gland cancer had the greatest SIR values and sustained risk with time: even after 10 years, a high relative risk was still present; SIR 15.3 (7.29–32.1) before 1 year and SIR 4.7 (1.18–18.8) after 10 years.

## DISCUSSION

This study confirms that there is a significantly increased relative risk of second primary cancers in individuals with SCC. The UV component of sunlight is widely believed to be the main risk factor for skin and lip cancer and this may explain the subsequent excess of malignant melanomas and SCCs of the lip.

It has been observed previously that the southern US (with higher exposure to UV radiation) has a significantly higher incidence of salivary gland cancer than the northern states (Spitz *et al*, 1988). In addition, it has been observed that there is an increased risk for melanoma and lip cancers subsequent to salivary gland cancer (Spitz *et al*, 1990). There is strong previous evidence for the association of skin cancers with non-Hodgkin's lymphoma and leukaemias, implying a causative role of UV light (Adami *et al*, 1995). Non-Hodgkin's lymphoma and SCC have been specifically associated, with the suggestion that UV radiation may have a suppressive effect on the immune system (Hall *et al*, 1995).

The great majority of the excess cancer occurrence in SCC is associated with smoking. This includes cancer of the lung, lip, salivary gland, oesophagus, larynx and pharynx, and leukaemia. This is consistent with the carcinogenic effects of smoking.

## Figures and Tables

**Table 1:** Standardised incidence ratios (SIR) and 95% confidence intervals for second cancers subsequent to SCC

	**Male**			**Female**		
**Site**	**Observed**	**Expected**	**SIR**	**Observed**	**Expected**	**SIR**
Lip	10	2.9	3.5 (1.9–6.4)	3	0.4	7.7 (2.5–24.0)
Salivary gland	36	3.3	11.0 (8.0–15.3)	9	0.9	10.6 (5.5–20.3)
Oropharynx	3	1.2	2.6 (0.8–8.0)	2	0.2	11.3 (2.8–45.4)
Hypopharynx	3	1.4	2.2 (0.7–6.8)	4	0.7	5.7 (2.1–15.3)
Oesophagus	73	58.0	1.3 (1.0–1.6)	14	15.8	0.9 (0.5–1.5)
Stomach	137	139.0	1.0 (0.8–1.2)	35	33.1	1.1 (0.8–1.5)
Colon	187	159.3	1.2 (1.0–1.4)	83	70.0	1.2 (1.0–1.5)
Liver	22	15.1	1.5 (1.0–2.2)	3	3.4	0.9 (0.3–2.7)
Pancreas	69	69.0	1.0 (0.8–1.3)	30	26.2	1.1 (0.8–1.6)
Nasal cavity and ear	4	1.9	2.1 (0.8–5.6)	4	0.6	6.8 (2.5–18.1)
Larynx	46	27.6	1.7 (1.2–2.2)	3	1.9	1.5 (0.5–4.8)
Bronchus and lung	710	558.6	1.3 (1.2–1.4)	95	80.2	1.2 (1.0–1.4)
Skin malignant melanoma	48	15.8	3.0 (2.3–4.0)	24	8.3	2.9 (1.9–4.3)
Other skin neoplasms (excl. BCC)	83	80.7	1.0 (0.8–1.3)	24	16.1	1.5 (1.0–2.2)
Breast	3	4.4	0.7 (0.2–2.1)	140	146.7	1.0 (0.8–1.1)
Prostate	389	385.6	1.0 (0.9–1.1)			
Bladder	154	167.4	0.9 (0.8–1.1)	25	22.5	1.1 (0.8–1.6)
Hodgkin's disease	6	4.3	1.4 (0.6–3.1)	1	1.5	0.7 (0.1–4.8)
Follicular NHL	6	2.2	2.7 (1.2–6.0)	5	1.1	4.7 (2.0–11.3)
Diffuse NHL	18	10.2	1.8 (1.1–2.8)	6	3.6	1.7 (0.7–3.7)
Peripheral/cutaneous T-cell lymphoma	10	2.3	4.4 (2.3–8.1)	1	0.5	2.0 (0.3–13.9)
Other NHL	77	33.7	2.3 (1.8–2.9)	21	12.4	1.7 (1.1–2.6)
Lymphoid leukaemia	41	24.8	1.7 (1.2–2.3)	12	6.8	1.8 (1.0–3.1)
Myeloid leukaemia	31	23.3	1.3 (0.9–1.9)	10	7.1	1.4 (0.8–2.6)
Total Number of all cancers incl. those not shown above	2567	2118.4	1.2 (1.2–1.3)	792	648.0	1.2 (1.1–1.3)
